# Systems level analysis of two-component signal transduction systems in *Erwinia amylovora*: Role in virulence, regulation of amylovoran biosynthesis and swarming motility

**DOI:** 10.1186/1471-2164-10-245

**Published:** 2009-05-26

**Authors:** Youfu Zhao, Dongping Wang, Sridevi Nakka, George W Sundin, Schuyler S Korban

**Affiliations:** 1 Department of Crop Sciences, University of Illinois at Urbana-Champaign, Urbana, IL 61801, USA; 2 Department of Plant Pathology, Michigan State University, East Lansing, MI 48824, USA; 3 Department of Natural Resources and Environmental Sciences, University of Illinois at Urbana-Champaign, Urbana, IL 61801, USA

## Abstract

**Background:**

Two-component signal transduction systems (TCSTs), consisting of a histidine kinase (HK) and a response regulator (RR), represent a major paradigm for signal transduction in prokaryotes. TCSTs play critical roles in sensing and responding to environmental conditions, and in bacterial pathogenesis. Most TCSTs in *Erwinia amylovora *have either not been identified or have not yet been studied.

**Results:**

We used a systems approach to identify TCST and related signal transduction genes in the genome of *E. amylovora*. Comparative genomic analysis of TCSTs indicated that *E. amylovora *TCSTs were closely related to those of *Erwinia tasmaniensis*, a saprophytic enterobacterium isolated from apple flowers, and to other enterobacteria. Forty-six TCST genes in *E. amylovora *including 17 sensor kinases, three hybrid kinases, 20 DNA- or ligand-binding RRs, four RRs with enzymatic output domain (EAL-GGDEF proteins), and two kinases were characterized in this study. A systematic TCST gene-knockout experiment was conducted, generating a total of 59 single-, double-, and triple-mutants. Virulence assays revealed that five of these mutants were non-pathogenic on immature pear fruits. Results from phenotypic characterization and gene expression experiments indicated that several groups of TCST systems in *E. amylovora *control amylovoran biosynthesis, one of two major virulence factors in *E. amylovora*. Both negative and positive regulators of amylovoran biosynthesis were identified, indicating a complex network may control this important feature of pathogenesis. Positive (non-motile, EnvZ/OmpR), negative (hypermotile, GrrS/GrrA), and intermediate regulators for swarming motility in *E. amylovora *were also identified.

**Conclusion:**

Our results demonstrated that TCSTs in *E. amylovora *played major roles in virulence on immature pear fruit and in regulating amylovoran biosynthesis and swarming motility. This suggested presence of regulatory networks governing expression of critical virulence genes in *E. amylovora*.

## Background

Prokaryotes use their small size and metabolic diversity to dominate every conceivable niche on earth. A large part of this success comes from the evolution of elaborate sensory systems to monitor and respond to dramatic fluctuations in their environment. Primary means of signal transduction in bacteria involve two-component signal transduction systems (TCSTs) [1,2]. The term "two-component" was coined in 1986 to describe a new class of regulatory systems found in bacteria; today, two-component systems represent major paradigms for signal transduction in prokaryotes and in lower eukaryotes [3,4]. The prototypical two-component system consists of a histidine kinase protein (HK) containing a conserved kinase core and a response regulator protein (RR) containing a conserved regulatory domain [4,5]. Extracellular stimuli are sensed by, and serve to modulate HK activities. The HK is autophosphorylated at a histidine residue, creating a high-energy phosphoryl group that is subsequently transferred to an aspartate residue in the RR via a reaction catalyzed by the RR itself. Phosphotransfer to the RR leads to activation of a downstream effector domain that elicits a specific response [1,4].

Two-component systems are distributed at varying frequencies among organisms of all domains, including Eubacteria, Archaea, and Eukarya [1,3]. The availability of complete genome sequences has allowed for a definitive assessment of the prevalence of two-component proteins. There are 30 HKs (five of which are hybrid kinases) and 32 RRs in *Escherichia coli *[6]. However, the number of two-component proteins differs greatly among bacteria. Often, parasitic bacteria encode fewer signaling proteins than relatively free-living bacteria [7]. Among sequenced plant pathogenic bacteria, the number of TCST genes is quite different. For example, the xylem-limited *Xylella fastidiosa *has the fewest TCST genes; whereas, the relatively adaptable *Pseudomonas syringae *and *Xanthomonas spp*. have the largest number of TCST genes [8,9]. Perhaps, the most attractive reason for studying two-component systems in bacteria is that TCST systems are used by pathogenic bacteria to control expression of virulence factors required for infection. Many such TCST systems are identified in both human and animal pathogens and in plant pathogens [7,10]. The *Agrobacterium tumefaciens *VirA/VirG system, the GacA/GacS of both *Pseudomonas *sp. and *Pectobacterium carotovora*, and the RpfCG of *Xanthomonas *spp. and *X. fastidiosa *are probably the most well-known and studied TCST systems involved in virulence gene expression in plant pathogens [10-12]. However, most of these studies focused on one or a few TCSTs. Only recently has genome-wide analysis of TCSTs in plant pathogenic bacteria become possible due to increased availability of whole genome sequences [8,9,13].

*E. amylovora *is the causal agent of fire blight, a devastating necrotic disease affecting apple, pear, and other rosaceous plants. Fire blight is one of the most important bacterial plant diseases worldwide that has a significant economic impact, resulting in crop losses of millions of dollars per year [14]. As a member of the *Enterobacteriaceae*, *E. amylovora *is related to many important human and animal pathogens such as *Es. coli*, *Salmonella enterica, Shigella flexneri, Yersinia enterocolitica*, and *Y. pestis*. Like many other Gram-negative plant pathogenic bacteria, *E. amylovora *utilizes both a type III secretion (T3SS) apparatus to deliver effector proteins into host plant cells, and the extracellular polysaccharide (EPS) amylovoran to cause disease [15-17]. In *E. amylovora*, structural components of T3SS encoded by the Hrp regulon are regulated by the two-component system HrpX and HrpY, which direct the expression of the NtrC family σ^54^-dependent, enhancer-binding protein HrpS [18]. Both HrpY and HrpS function in the activation of expression of the alternate sigma factor HrpL, thereby regulating various genes and operons of the Hrp regulon. Expression of *hrpX *and *hrpS *is regulated by low pH, low nutrients, and low temperature conditions, mimicking the plant apoplast [18]. The biosynthesis of amylovoran is regulated by another TCST system, the RcsCDB phosphorelay system [17]. Recently, we identified several TCST genes including *hrpX, grrS*, and *envZ *that are induced during infection of host tissue in *E. amylovora*, indicating that TCSTs are key players in controlling the expression of virulence factors required for infection, and thus we hypothesize that there are likely networks controlling virulence gene expression [19]. However, other TCSTs in *E. amylovora *have either not been identified or have not yet been studied. This prompted us to use a systems approach to explore the function of TCSTs in *E. amylovora*. Understanding the genetics and molecular mechanisms of *E. amylovora *signaling will greatly enhance the likelihood of developing novel methods of controlling the disease.

The EnvZ-OmpR and GrrS-GrrA (also called GacSA, BarA-UvrY) are two widely-distributed and well-studied TCSTs in γ-proteobacteria. They represent those paradigms of signal transduction systems having pleiotropic effects, thus suggesting both systems are global regulators [2,20]. EnvZ is a transmembrane sensor that predominantly responds to acidic pH conditions and changes in osmolarity, and subsequently phosphorylates OmpR. The EnvZ-OmpR system has been originally reported to govern the expression of *ompC *and *ompF *genes, encoding two major outer-membrane porins [21]. In addition to its role in porin osmoregulation, OmpR is involved in regulating various cellular components including flagellar gene expression, fatty acid transport, curli fibre formation, and cell division as a dual regulator; i.e., negative or positive [22]. In *Salmonella *spp., OmpR-EnvZ regulates another TCST system SsrA-SsrB, that in turn regulates the T3SS produced by *Salmonella *pathogenicity island 2 (SPI-2) [23-26]. OmpR also negatively regulates expression of invasin in *Yersinia enterocolitica *and the T3SS in *P. syringae *[27,28].

GacS and GacA homologs have been identified in many Gram-negative bacterial genera, including *Azotobacter, Erwinia, Escherichia, Legionella, Pectobacterium, Pseudomonas, Salmonella, Serratia*, and *Vibrio *[12,20,29]. The sensor kinase GacS was initially discovered as a key regulator of virulence in the plant pathogen *P. syringae *[see review [12]]. The GacSA system has since been reported to regulate an array of phenotypes, including biofilm formation, alginate biosynthesis, production of toxins and extracellular enzymes [30,31], proteases, siderophores, swarming motility [32], and type III secretion [10,29,33-35]. Similar to EnvZ-OmpR, GacSA also has a dual regulatory function; i.e., as a positive or negative regulator. The GacS/GacA (BarA/UvrY) system positively controls the expression of one to five genes specifying small RNAs (sRNAs), thus upregulating the productions of proteins that are otherwise repressed by small RNAs (RsmA/CsrA) [32,36]. Most *gacS/gacA *mutants demonstrate reduced production of virulence factors and reduced virulence in a variety of host-pathogen systems [12,20]. An alternative scenario is that the GacSA cascade downregulates the expression of flagellar genes in *P. fluorescens *or *Es. coli *[20].

In this study, our genome-wide analysis, using the recently closed genome sequence of *E. amylovora *Ea273  identified a total of 46 TCST genes in *E. amylovora *including 17 sensor kinases, 20 response regulators, three hybrid HKs, four RRs with enzymatic output domain (EAL-GGDEF proteins), and two kinases. A comparative genomic analysis of TCSTs was then conducted in related enterobacteria enabling classification of the TCSTs in *E. amylovora*. A total of 59 deletion mutants were generated, and their contribution to virulence, amylovoran biosynthesis, and swarming motility was characterized. Our findings suggested that TCSTs in *E. amylovora *played a major role in virulence and in the regulation of amylovoran biosynthesis and swarming motility. This indicated that networks of gene regulation existed in *E. amylovora *that respond to different environmental and host signals.

## Results

### Identification and comparison of TCSTs in *E. amylovora*

We utilized two approaches to identify HKs and RRs in the genome of *E. amylovora*. First, using a candidate gene approach, known HK and RR sequences from *Es. coli *K12 and *P. carotovora *pv. *atroseptica *SCRI1043 were used to search the closed genome sequence of *E. amylovora *. Second, based on conserved domains of known HKs and RRs, other putative HKs and RRs in *E. amylovora *were identified and confirmed by searching the complete genome sequences for proteins containing HK and RR domains using Pfam Hidden Markov Model (HMM) profiles and BLASTP. As a result, we identified 46 TCST and other related signal transduction genes in *E. amylovora *(Table 1 and Table S1, [see Additional file 1]), excluding two sets of chemotaxis genes (*cheABRWYZ*). These putative regulatory genes represented 1.36% of the genome (3367 genes) [37]. Among them, 17 are sensor kinases, three hybrid HKs, 20 DNA-or ligand-binding RRs, four RRs with enzymatic output domain (EAL-GGDEF proteins), and two putative kinases (Table 1).

**Table 1:** TCSTs and other signal transduction genes in *Erwinia amylovora *and mutant construction

**Gene/Operon^a^**	**HK/RR/Hybrid/kinase^b^**	**Mutants constructed^c^**
*arcB, arcA*	HK, RR	ΔarcB, ΔarcA
*baeSR*	HK, RR	ΔbaeS, ΔbaeR, ΔbaeSR
*cpxA1R1*	HK, RR	ΔcpxA1, ΔcpxR1, ΔcpxAR1
*cpxA2R2*	HK, RR	ΔcpxA2, ΔcpxR2, ΔcpxAR2
*dcuSR*	HK, RR	ΔdcuS, ΔdcuR, ΔdcuSR
*envZ/ompR*	HK, RR	ΔenvZ, ΔompR, ΔenvZ/ompR
*grrS, grrA*	HK hybrid, RR	ΔgrrS, ΔgrrA
*hrpXY, hrpS*	HK, RR, EBP	ΔhrpX, ΔhrpY, ΔhrpXY, ΔhrpXYS
*kdpD, kdpE*	HK, RR	ΔkdpD, ΔkdpE
*narQP*	HK, RR	ΔnarQ, ΔnarP, ΔnarQP
*phoQP*	HK, RR	ΔphoQ, ΔphoP, ΔphoQP
*phoRB*	HK, RR	ΔphoR, ΔphoB, ΔphoRB
*pmrBA*	HK hybrid, RR	ΔpmrB, ΔpmrA, ΔpmrBA
*rcsCDB*	HK hybrid, HPT, RR	ΔrcsD, ΔrcsB, ΔrcsC, ΔrcsBD
*rstB, rstA*	HK, RR	ΔrstB, ΔrstA
*yehUT*	HK, RR	ΔyehU, ΔyehT, ΔyehUT
*yfhKA*	HK, RR	ΔyfhK, ΔyfhA, ΔyfhKA
**Other signal transduction genes used in this study^b^**		
*eamIR*	AHL, RR	ΔeamI, ΔeamR
*luxQ, luxP*	AI2 sensor kinase, RR	ΔluxQ, ΔluxP
*Spk1*	Serine kinase	Δspk1
*ybjN*	Kinase	ΔybjN
*yciR*	GGDEF-EAL	ΔyciR
*yddV*	GGDEF	ΔyddV
*yegE*	GGDEF	ΔyegE
*yoaD*	EAL	ΔyoaD

^a ^Genes for one TCST system are listed separately if they are not encoded within an operon in the genome. Otherwise, TCSTs are listed as an operon if genes are present together. Mutants for *ntrBC *were not constructed.

^b ^All TCSTs are sensor kinases and their corresponding DNA-binding response regulators. We included two sets of quorum sensing genes and four RRs with enzymatic output domain (EAL-GGDEF proteins) and two kinases. EBP: NtrC family of enhancing-binding protein. HPT: Histidine phosphotransfer domain; AHL: Acyl-homoserine lactone; GGDEF: Diguanylate cyclase; EAL: Type I c-di-GMP phosphodiesterase.

^c ^Mutant designation see Table 3.

When compared to TCSTs in other enterobacteria, the majority of TCSTs in *E. amylovora *have counterparts in related enterobacteria, including *Dickeya dadantii *(*Erwinia chrysanthemi*) 3937, *P. carotovora *subsp. *atroseptica *SCRI1043, *Erwinia tasmaniensis *Et1/99, and *Es. coli *K12 (Table 2 and Table S1, [see Additional file 1]). Only one HK and RR pair (*ypdAB*) in *E. tasmaniensis *is not present in *E. amylovora *Ea273 (Table 2). The number of TCSTs in *E. amylovora *is the lowest among other sequenced related plant pathogenic enterobacteria and *Es. coli *(Table S1, [see Additional file 1]). BLAST searches revealed that the HKs and RRs in *E. amylovora *shared the highest amino acid (aa) identity/similarity to those of *E. tasmaniensis *Et1/99, an epiphytic bacterium isolated from apple flowers in Australia [38], except for HrpX/Y which shared the highest aa identity/similarity to those in *Erwinia pyrifoliae*, a related blight pathogen of Asian pear (Table 2). It is interesting to note that HrpXY, which regulates T3SS gene expression in plant pathogenic enterobacteria, is the only TCST that is not present in other mammalian enterobacterial pathogens [39].

**Table 2:** Comparison of *E. amylovora *and *E. tasmaniensis *TCST and other signal transduction genes^a^

**Gene^b^**	**Length of deduced Amino acid^c^**	**aa Identity/Similarity (%)^d^**	**Gene^b^**	**Length of deduced Amino acid^c^**	**aa Identity/Similarity (%)^d^**
*arcB*	779	93/95	*arcA*	238	96/99
*baeS*	461	89/93	*baeR*	235	88/94
*grrS *(*barA*)	909	91/94	*grrA *(Eta_20780)	219	94/98
*cpxA1*	459	96/98	*cpxR1*	233	95/97
*cpxA2 *(Eta_10960)	450	82/88	*cpxR2 *(Eta_10950)	226	88/93
*dcuS*	546	79/86	*dcuR*	239	86/93
*envZ*	449	95/97	*ompR*	239	100/100
*hrpX*	494	89/93	*hrpY*	213	94/96
*kdpD*	890	95/97	*kdpE *(Eta_23200)	226	89/92
*luxQ *(Eta_11320)	419 (420)	89/93	*luxP *(Eta_11350)	258	74/89
*narQ*	330	N/A	*narP*	209 (210)	71/83
*ntrB*	349	96/98	*ntrC*	469	98/98
*phoQ*	481	92/96	*phoP*	222	95/98
*phoR*	437	88/92	*phoB*	229	96/98
*pmrB*	350	81/89	*pmrA*	219	94/98
*rcsC*	885 (890)	88/93	*rcsB*	215	99/100
*rcsD *(*yojN*)	987 (951)	84/91	*eamR *(*sdiA*)	240	84/92
*rstB*	426	92/96	*rstA*	242	93/97
*ybjN *(Eta_21780)	159	91/96	*Spk1 *(Eta_06360)	468	91/92
*yddV *(hmsT)	365	81/88	*yegE*	848 (877)	78/88
*yehU*	564 (562)	84/90	*yehT*	239	80/89
*yfhK*	463 (475)	87/91	*yfhA *(Eta_10100)	444	93/96
*yoaD *(rtn)	516 (523)	81/89	*yciR *(Eta_30620)	818 (820)	82/91

^a ^Sequences for *E. amylovora *TCST genes are from the whole genomic sequence of *E. amylovora *. One TCTS system (*ypdAB*) in *E. tasmaniensis *is not present in *E. amylovora *Ea273. Two sets of CheABRWYZ exist in *E. amylovora*, but only one set in *E. tasmaniensis*.

^b ^In parentheses is gene or gene number for the corresponding gene in *E. tasmaniensis*.

^c ^The number in parentheses is the length of deduced amino acid for the corresponding protein in *E. tasmaniensis *which differs from those in *E. amylovora*.

^d ^All data are the highest aa similarity and identify found in NCBI database as of today except for HrpXY which share the highest aa identity/similarity to those in *E. pyrifoliae *(98/99%; 99/99%, respectively). N/A: not available.

### Classification of HKs and RRs in *E. amylovora*

In general, HKs are highly variable in amino acid sequence length; whereas, RRs are highly conserved in their receiver domain (REC). Classification of HKs is usually based on alignment of residues surrounding the H-box that contains the conserved His residue within the histidine kinase domain (HisKA) [9,40]. Multiple sequence alignment using Clustal X has classified the HKs of *E. amylovora *into four groups [40]. Among them, 15 belong to groups IA (11), IB (3), and IC (1); and one (DcuS), two (NarQ and HrpX), and one (YehU) belong to groups II, III, and IV HKs, respectively (Table S1, [see Additional file 1] and Figure S1, [see Additional file 2]). This is the first time that HrpX has been classified as a type III HK. However, RcsD (also known as YojN), containing a histidine phosphotransfer (HPt) domain, but lacking a conserved histidine residue in the HisKA domain, could not be classified.

Analysis of the domain architecture of *E. amylovora *HKs, by either searching the Conserved Domain Databases (CDD) [41] or using the SMART program [42], has revealed that all HKs in *E. amylovora*, except for HrpX and NtrB, contain one to three hydrophobic transmembrane (TM) helices within the N-terminal sensor region. This indicates that these HKs are periplasmic sensors. HrpX and NtrB contain two and one PAS domain (initially found in *P*ER, *A*RNT, and *S*IM proteins), respectively, within the N-terminal sensor region, indicating that they are soluble and cytoplasmic proteins that sense intracellular signals [18]. The YehU contains a 5TMR-LYT (5 TM receptor domain, LytS-like) sensory input domain before the two TM helices, thus indicating that the YehU is an intramembrane sensor.

Often, RRs are classified into families based on their output domains and domain combinations [43]. By searching the SMART program and conducting a structural similarity of C-terminal output domains [4,43], it is revealed that 20 RRs contain either DNA- or ligand-binding output domains in *E. amylovora *(Table S1, [see Additional file 1]). Among them, eleven, four, two, and one are further classified into OmpR-, NarL-, NtrC-, and LytR-like proteins, respectively (Table S1, [see Additional file 1]). These RRs belong to four major families of RRs found in prokaryotes and account for about 64% of all RRs identified thus far [43]. In addition, four RRs (YciR, YddV, YegE, and YoaD) contain enzymatic output domains including GGDEF and EAL domains (Table 1). The GGDEF and EAL domains have diguanylate cyclase and Type I c-di-GMP phosphodiesterase activity, respectively, and these proteins are involved in regulating virulence functions in some plant pathogens [44].

### Systematic deletion of TCSTs and related signaling genes, and their roles in virulence on immature pear fruit

To pursue comprehensive identification of two-component signaling pathways required for virulence, swarming motility, and amylovoran biosynthesis in *E. amylovora*, deletion mutants were generated for those genes/operons identified above, and also included *hrpS *and *eamI*. Deletions were made using the Lambda-Red recombinase technique, as previously described for *Es. coli *and *E. amylovora *[45-47]. A total of 59 single-, double- and triple- stable deletion mutants were generated (Table 1 and Table 3). For these mutants, the majority of the coding region of each gene or operon was deleted and replaced by the Cm^R ^or Km^R ^marker gene, except for the first and last 50 nt of the gene or operon, thus minimizing any polar effects of the mutation.

**Table 3:** *E. amylovora *TCTS mutants and their phenotypes

**Gene/Operon^a^**	**Mutant designation**	**Virulence assay^b^**	**Amylovoran production (48 h) (OD_600_)^c^**	**Significance level^e^**	**Distance of swarming motility (48 h, cm)^d^**		**Significance level^e^**
WT		+++	0.08 ± 0.002	nop	C	2.7 ± 0.15	cde
*ams*	Z0118Δams	---	0.002 ± 0.0005	r	ND	ND	ND
*flhD*	Z2946ΔflhD	+++	ND	ND	C	0.7 ± 0	w
*fliA*	Z2986ΔfliA	+++	ND	ND	C	0.7 ± 0	w
*arcA*	Z1306ΔarcA	+++	0.053 ± 0.0001	qr	C	1.23 ± 0.09	uv
*arcB*	Z0117ΔarcB	+++	0.067 ± 0.0002	pq	C	1.83 ± 0.28	qr
*baeR*	Z3120ΔbaeR	+++	0.14 ± 0.045	klmn	I	1.87 ± 0.25	qr
*baeS*	Z3119ΔbaeS	+++	0.13 ± 0.03	klmn	I	2.07 ± 0.06	nopq
*baeSR*	Z3219–20ΔbaeSR	+++	0.1 ± 0.01	lmn	I	2.07 ± 0.06	nopq
*cpxA1*	Z0686ΔcpxA1	+++	0.09 ± 0.014	nop	I	1.40 ± 0.22	tu
*cpxA1R1*	Z0686–87ΔcpxAR1	+++	0.055 ± 0.003	pqr	I	1.33 ± 0.05	uv
*cpxR1*	Z0687ΔcpxR1	+++	0.08 ± 0.008	nop	I	1.16 ± 0.13	v
*cpxA2*	Z3367ΔcpxA2	+++	0.35 ± 0.023	hgi	I	2.5 ± 0.4	efghi
*cpxA2R2*	Z3367–68ΔcpxAR2	+++	0.29 ± 0.07	i	I	2.07 ± 0.34	opqr
*cpxR2*	Z3368ΔcpxR2	+++	0.35 ± 0.058	hgi	I	2.5 ± 0.14	efgh
*dcuR*	Z2361ΔdcuR	+++	0.198 ± 0.01	k	I	2.2 ± 0.17	ijklm
*dcuS*	Z2362ΔdcuS	+++	0.171 ± 0.02	kl	I	2.07 ± 0.32	ijklm
*dcuSR*	Z2361–62ΔdcuSR	+++	0.256 ± 0.02	j	I	2.23 ± 0.38	mnop
*eamI*	Z2195ΔeamI	+++	0.256 ± 0.046	j	I	2.23 ± 0.12	ijklm
*eamR*	Z2194ΔeamR	+++	0.544 ± 0.1	de	I	2.03 ± 0.31	pqr
*envZ*	Z0270ΔenvZ	+++	1.55 ± 0.06	c	C	1.03 ± 0.11	v
*envZ/ompR*	Z0270–71 ΔenvZ/ompR	+++	1.7 ± 0.05	a	C	1.03 ± 0.11	v
*ompR*	Z0271ΔompR	+++	1.63 ± 0.06	b	C	1.03 ± 0.11	v
*grrA*	Z2198ΔgrrA	+++	1.58 ± 0.15	bc	C	3.15 ± 0.07	a
*grrS*	Z3742ΔgrrS	+++	1.56 ± 0.09	c	C	3.2 ± 0.05	a
*hrpX*	Z3962ΔhrpX	+++	0.69 ± 0.11	d	I	1.73 ± 0.15	rs
*hrpY*	Z3963ΔhrpY	+++	0.471 ± 0.05	e	I	2.0 ± 0.0	opqr
*hrpXY*	Z3962–63 ΔhrpXY	+++	0.45 ± 0.09	ef	I	2.33 ± 0.15	hijkl
*hrpXYS*	Z3962–64 ΔhrpXYS	---	0.4 ± 0.09	gh	I	2.27 ± 0.15	hijkl
*kdpD*	Z0414ΔkdpD	+++	0.372 ± 0.02	hgi	I	2.13 ± 0.12	nopq
*kdpE*	Z1912ΔkdpE	+++	0.09 ± 0.035	no	I	1.43 ± 0.19	tu
*luxP*	Z3324ΔluxP	+++	0.419 ± 0.12	g	I	1.87 ± 0.23	rs
*luxQ*	Z3327ΔluxQ	+++	0.625 ± 0.11	de	I	1.9 ± 0.17	qr
*narP*	Z3390ΔnarP	+++	0.1 ± 0.001	lmn	I	2.2 ± 0.0	klmno
*narQ*	Z3389ΔnarQ	+++	0.08 ± 0.003	lmn	I	2.4 ± 0.1	fghij
*narQP*	Z3389–90 ΔnarQP	+++	0.08 ± 0.002	nop	I	2.27 ± 0.25	ghijkl
*phoB*	Z1675ΔphoB	+++	0.145 ± 0.003	klmn	I	2.6 ± 0.2	defg
*phoR*	Z1676ΔphoR	+++	0.366 ± 0.01	hgi	I	2.75 ± 0.15	cd
*phoRB*	Z1675–76 ΔphoRB	+++	0.312 ± 0.01	hi	I	2.7 ± 0.1	cde
*phoP*	Z2324ΔphoP	+++	0.15 ± 0.02	klm	I	2.45 ± 0.17	cdef
*phoQ*	Z2323ΔphoQ	+++	0.106 ± 0.01	lmno	I	2.25 ± 0.23	efghi
*phoQP*	Z2323–24 ΔphoQP	+++	0.18 ± 0.002	jk	I	2.25 ± 0.15	ijklm
*pmrA*	Z0089ΔpmrA	+++	0.05 ± 0.02	pqr	I	2.3 ± 0.19	hijkl
*pmrB*	Z0090ΔpmrB	+++	0.10 ± 0.01	lmno	I	2.40 ± 0.17	defgh
*pmrBA*	Z0089–90 ΔpmrBA	+++	0.07 ± 0.01	opq	I	2.3 ± 0.20	fghij
*rcsB*	Z3206ΔrcsB	---	0.002 ± 0.0005	r	I	2.51 ± 0.17	efghi
*rcsC*	Z3207ΔrcsC	---	0.4 ± 0.04	fg	I	2.45 ± 0.2	fghij
*rcsD *(*yojN*)	Z3205ΔrcsD	---	0.002 ± 0.0002	r	I	2.48 ± 0.28	efghi
*rcsDB*	Z3205–06 ΔrcsBD	---	0.003 ± 0.0001	r	I	2.43 ± 0.11	fghijk
*rstA*	Z2651ΔrstA	+++	0.359 ± 0.045	hgi	I	1.6 ± 0	st
*rstB*	Z2662ΔrstB	+++	0.1 ± 0.032	lmn	I	2.03 ± 0.06	opqr
*spk1*	Z1250Δspk1	+++	0.17 ± 0.04	kl	I	2.58 ± 0.14	cdef
*ybjN*	Z2074ΔybjN	+++	1.65 ± 0.12	a	I	2.05 ± 0.26	lmno
*yciR*	Z0955ΔyciR	+++	0.111 ± 0.0012	lmno	I	1.9 ± 0.08	pqr
*yddV*	Z1215ΔyddV	+++	0.043 ± 0.001	qr	C	2.9 ± 0.14	bc
*yegE*	Z3113ΔyegE	+++	0.22 ± 0.007	j	I	2.9 ± 0.14	bc
*yehT*	Z3996ΔyehU	+++	0.186 ± 0.026	k	I	2.5 ± 0.2	efghi
*yehU*	Z3995ΔyehT	+++	0.15 ± 0.04	klm	I	2.33 ± 0.21	fghij
*yehUT*	Z3995–96 ΔyehUT	+++	0.15 ± 0.004	klm	I	2.4 ± 0.17	fghij
*yfhA*	Z3498ΔyfhA	+++	0.07 ± 0.012	opq	I	2.3 ± 0.1	hijkl
*yfhK*	Z3495ΔyfhK	+++	0.06 ± 0.002	pq	I	2.33 ± 0.25	fghij
*yfhKA*	Z3495–98 ΔyfhKA	+++	0.062 ± 0.0005	pq	I	2.17 ± 0.12	lmno
*yoaD*	Z0355ΔyoaD	+++	0.22 ± 0.008	j	I	2.4 ± 0.37	ghijkl

^a ^ams operon encodes amylovoran biosynthesis. The entire operon (*amsA-L*; 15.8 kb) was deleted in the *ams *mutant [47]. *flhD *and *fliA *encode two regulators of flagella biosynthesis. These three mutants were used as negative controls for amylovoran biosynthesis and swarming motility, respectively.

^b ^Virulence assay was performed using immature pear fruits as described. +++: pathogenic as WT; ---: non-pathogenic.

^c ^Amylovoran production was measured 48 hr post inoculation. ND: not determined.

^d ^Distance of swarming motility was measured 48 hr post inoculation. Original circle was about 0.6 cm in diameter. I: irregular movement; C: circular. ND: not determined.

^e ^Values within the amylovoran production and distance of swarming motility columns followed by the same letter are not significantly different according to one way ANOVA and student-Newmans-Kleus test (*P *= 0.05).

For prokaryotes, genes for cognate pairs of HKs and RRs are typically encoded together in a single operon [1]. Because of this unique feature for HKs and RRs, and the potential cross-talk between different HKs and RRs, these single deletion mutants generated in this study have been designed to have little or no effect on expression/function of the cognate gene in the operon. Furthermore, operon deletion mutants have been generated so that phenotypes of single gene deletion mutants could be compared with those of operon deletion mutants to determine whether or not there is cross-talk between HKs and RRs. All mutants and their designations are listed in Table 3.

To determine the role of deletion mutants in *E. amylovora *virulence, an assay, routinely used to evaluate virulence of *E. amylovora *WT strains, of all 59 TCST mutants on immature pear fruits was conducted [19,46,48]. Mutants and WT strains were inoculated on immature pears as described, and disease development was assessed for up to eight days. Results showed that five mutants, including three single (*rcsB*, *rcsC*, *rcsD *mutant), one double (*rcsBD*), and one triple mutant (*hrpXYS*), were non-pathogenic on immature pears (Table 3). However, *hrpX*, *hrpY*, and *hrpXY *mutants remained to exhibit full-virulence, as that of WT, on immature pear fruit (Table 3). Previous genetic studies have shown that a mutation in *rcsB *renders *E. amylovora *nonpathogenic and abolishes amylovoran production [49]. Our results further demonstrated that the RcsCDB phosphorelay system was essential for virulence in *E. amylovora *[17]. In addition, all other mutants induced similar symptoms to the WT strain on immature pear fruit (Table 3).

### Phenotypic analysis of deletion mutants

**Figure 1:**
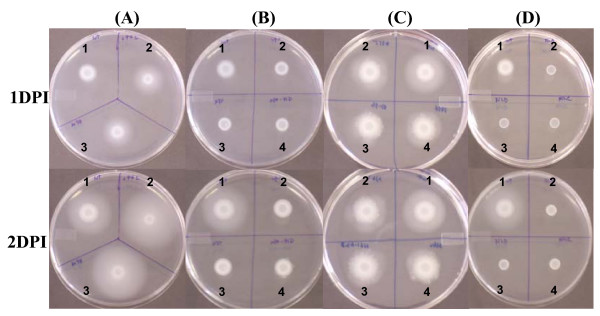
**Comparison of motility on swarming plates for WT and TCST mutants**. Bacterial strains were spotted on the swarming plate (0.3% agar) and incubated at 28°C. Photos were taken at one or two days post inoculation. DPI: days post inoculation. **A1 to D1**: WT strain; **A2**: *grrS *mutant; **A3**: *grrA *mutant; **B2**: *envZ *mutant; **B3**: *ompR *mutant; **B4**: *envZ/ompR *double mutant; **C2 to C4**: representative mutants with irregular movements at two days post inoculation; **D2 to D4**: *flhD*, *flhC *and *fliA *mutant, respectively. Flagella mutants were used as negative controls.

**Figure 2:**
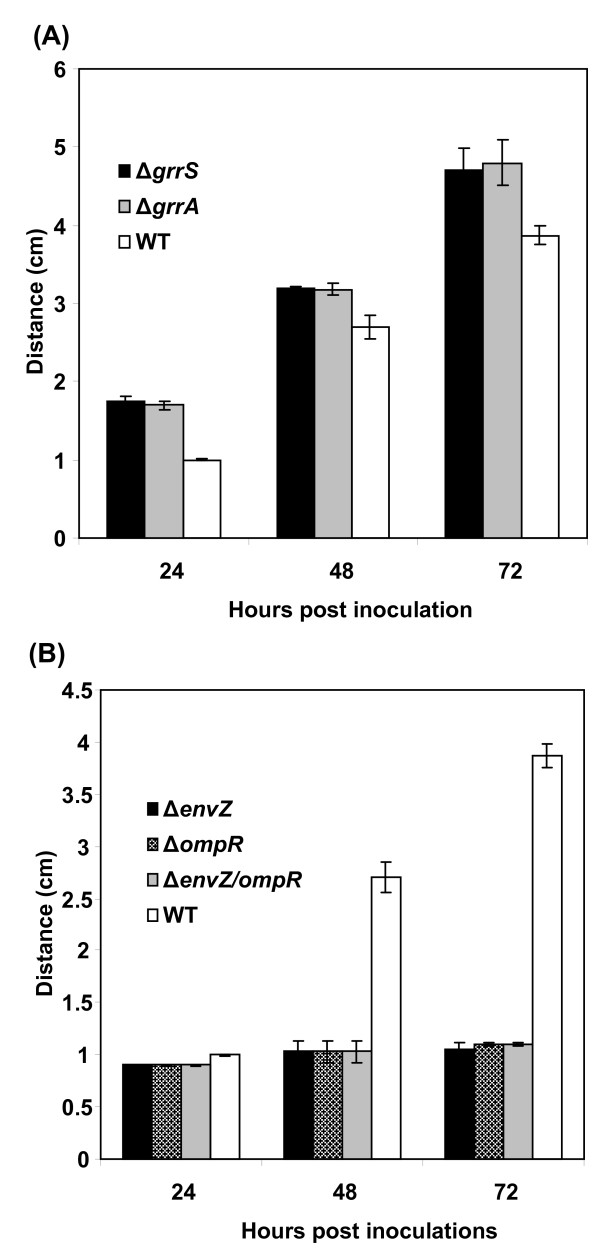
**Comparison of the swarming distance of WT and TCST mutants**. The diameters of the swarming circle were measured 24, 48 and 72 hrs after incubation. The experiments were repeated at least three times.

All generated mutants did not exhibit any distinctive phenotypes on rich LB medium, except for a single mutant which was mucoid (*ybjN *mutant). Therefore, phenotypes of the 59 deletion mutant strains were evaluated using a swarming plate assay [50,51]. WT cells can swarm via the combined effects of flagellar motility, chemotaxis, and growth, thus creating a circular colony. Defects in cell motility, chemotaxis, or growth can produce alterations in swarming size or density [51]. For *E. amylovora *WT and mutant strains, bacterial suspensions were plated onto swarming agar plates containing 0.3% agar, as previously described [50,51]. Swarming diameter and density were determined following incubation at 28°C for up to 72 hr, and three different swarming phenotypes were identified. Two mutants, *grrA *and *grrS*, exhibited substantially larger and lower density swarms than the WT (Figure 1A; Table 3, significance level a). In addition, three mutants, *envZ*, *ompR*, and *envZ/ompR *double-mutant, showed dramatic reduction in swarm size, but with increased density (Figure 1B; Table 3, significance level v). All these five mutants exhibited circular swarming (Figure 1A and 1B). A third group of mutants showed smaller swarms, colonies exhibited irregular circular patterns, and these included *arcAB*, *baeRS*, *cpxA1R1*, *kdpE*, *luxPQ*, *rstAB*, and *yciR *mutants (Figure 1C; Table 3, significance levels o to u). Although the distance of the *cpxA1R1 *mutants had similar significant level as that of *envZ/ompR *mutants at 48 hr, these mutants were included in the third group due to its irregular movements and with increased distance at 72 hr in contrast to *envZ/ompR *mutants which remained the same at 72 hr (Figure 2B). The rest of mutants showed even less change in swarm distance. As negative controls, all flagella-deficient mutants, *flhDC *and *fliA*, were non-motile on swarming plates (Figure 1D).

As shown in Figure 1A, *grrS/grrA *mutant strains showed precocious swarming and moved faster than the WT strain, with a diameter of 1.7 cm within 24 hr compared to 1 cm for the WT strain, from an original spot of 0.6 cm in diameter (Figure 2A). The swarming distance was 3.2 and 4.8 cm for *grrS/grrA *mutants and 2.7 and 3.8 cm for WT strain, respectively, at 48 and 72 hr (Figure 2A). When *grrS *and *grrA *mutants were complemented with their own gene cloned from the WT strain, the hyper-motile phenotype was restored in these mutants with a swarming distance, ~2.8 cm after 48 hr of incubation, similar to that of the WT strain (data not shown). In contrast, *envZ/ompR *mutants showed reduced swarming even after 72 hr (Figure 2B), and colonies exhibited more dense and fuzzy appearance. The diameter for *envZ/ompR *mutants was about 0.8, 1.0, and 1.0 cm for 24 to72 hr, from an original spot of 0.6 cm in diameter, respectively (Figure 2B). When *envZ/ompR *mutants were complemented by their own gene cloned from the WT strain, the 'low-motile' phenotype was partially restored. The diameter (about 1.5 cm after 48 hr of incubation) for the complemented mutant strains was comparable to that of the WT strain (Data not shown). However, the swarming phenotype remained different between *envZ/ompR *mutants and those of *flhDC *and *fliA *mutants, which exhibited no movement and maintained the original size of 0.6 to 0.7 cm in diameter (Figure 1BD). In summary, these results demonstrated that EnvZ/OmpR acts as a positive regulator of swarming motility, while GrrS/GrrA is a negative regulator of swarming motility in *E. amylovora*. These findings demonstrated that additional TCST genes might also control swarming motility in *E. amylovora*.

### Regulation of amylovoran biosynthesis

**Figure 3:**
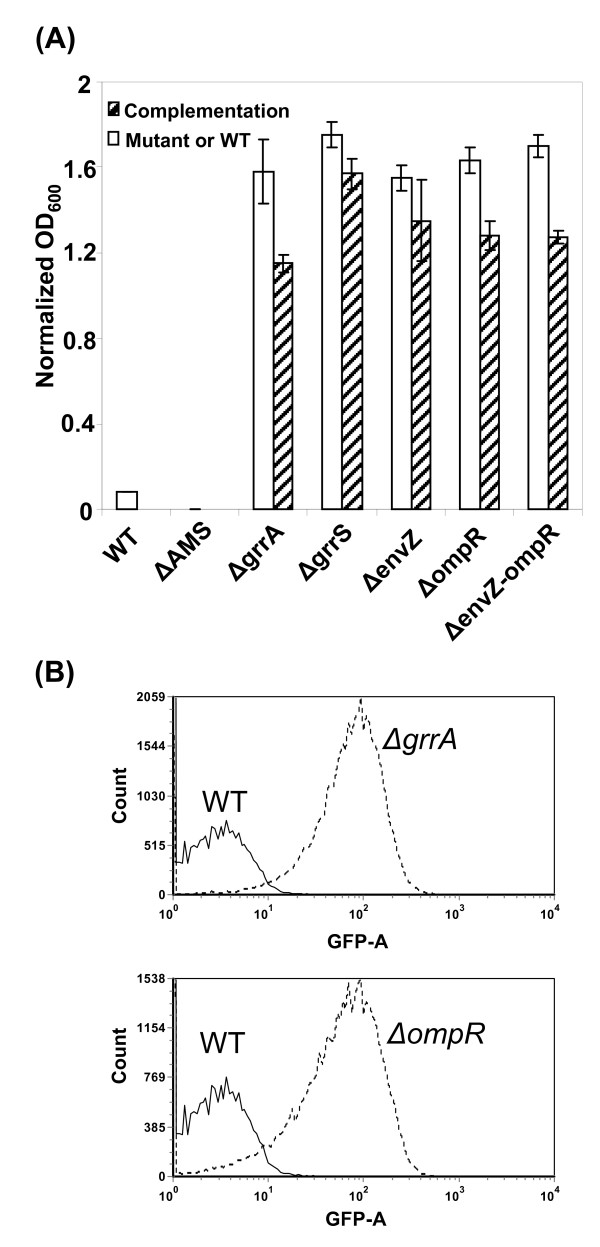
**TCSTs regulate amylovoran biosynthesis and gene expression**. **(A) **Amylovoran production of *E. amylovora *WT and TCST mutants *in vitro*. Bacterial strains were grown in MBMA media with 1% sorbitol for 48 hrs at 28°C with shaking. The amount of amylovoran was measured with the CPC assay and normalized to a cell density of 1. Amylovoran operon (*amsA-L*) deletion mutant (Δ*ams*) was used as a negative control [47]. **(B) **Gene expression of the *amsG *gene in WT and TCST mutants *in vitro*. GFP intensity in WT and TCST mutants containing *amsG *promoter-*gfp *fusion plasmid was measured by flow cytometry. GFP-A: Green fluorescence protein absorbance; Count: Number of cells.

Based on the virulence assay, the RcsCDB system that regulates amylovoran biosynthesis is essential for virulence in *E. amylovora*. Phenotypes of the 59 deletion mutant strains were further evaluated by measuring amylovoran biosynthesis *in vitro*. The amylovoran concentration in the supernatant of bacterial cultures was quantitatively determined using a turbidity assay with cetylpyrimidinium chloride (CPC), as previously described [52]. In this screening, four groups of mutants exhibiting varying levels of amylovoran production *in vitro *were identified (Table 3). For Group I, three mutants (*rcsB*, *rcsD, and rcsBD*) exhibited lack of amylovoran production (Table 3, significance level r); For Group II, six mutants (*grrA*, *grrS, ybjN, envZ*, *ompR*, and *envZ/ompR *double-mutant) showed a dramatic increase in amylovoran production, about 20-fold, compared to that of the WT (Figure 3A; Table 3, significance levels a to c); Group III mutants demonstrated increased production of amylovoran, from 2- to 8-fold, compared to that of the WT, and these included *hrpX*, *hrpY*, *hrpXY*, *hrpXYS*, *rcsC*, *luxPQ*, and *eamIR *(Table 3, significance levels d to j). Group IV mutants did not show much difference in amylovoran production when compared to that of WT, and these included *pmrAB*, *narPQ*, and *yfhAK *(Table 3, significance levels k to p). As a negative control, an amylovoran operon deletion mutant, *ams*, lacked amylovoran production (Figure 3A; Table 3, significance level r) [17,47].

For group I mutants, we have recently described how the RcsCDB phosphorelay system regulates amylovoran biosynthesis [17]. This study focused on group II mutants, except for the *ybjN *mutant, which will be reported in the future. Amylovoran production of five mutants, including *grrA*, *grrS, envZ*, *ompR*, and the *envZ/ompR *double-mutant, was partially complemented by their own gene/operon cloned from the WT strain. As shown in Figure 3A, complemented strains produced slightly less amylovoran than that of the mutant strains. To correlate amylovoran production with amylovoran biosynthesis gene expression [17], the level of expression of the *amsG *gene, the first gene in the *ams *(amylovoran) operon, was measured using GFP as a reporter in two RR mutants (*grrA *and *ompR*). GFP intensity was measured in the WT and in mutants containing the *amsG *promoter-GFP fusion using flow cytometry [17]. The *amsG *gene was expressed at a basal level in the WT strain, with a GFP intensity value of 1.7 (geometric mean), compared to a geometric mean value of 1.5 for the control vector. The geometric mean value of the GFP intensity of the *amsG *promoter was 26.2 and 60.5 for *ompR *and *grrA *mutants, respectively (Figure 3B). These results indicated that amylovoran production was negatively regulated by both EnvZ/OmpR and GrrA/S systems, and a regulatory network might be regulating amylovoran production in *E. amylovora*.

## Discussion

In this study, 46 TCSTs have been identified in *E. amylovora*, a pathogen of rosaceous plants which mainly resides in the plant xylem, but can also grow epiphytically on stigmas of flowers. Compared to other plant pathogenic enterobacteria such as *D. dadantii *and *P. carotovora *subsp. *atroseptica*, both of which can survive not only in plants, but also in soil, or the animal counterpart *Es. coli*, the number of TCSTs present in *E. amylovora *is relatively small, thus may reflecting the particular host niche for this pathogen. Furthermore, the genome size (3.9 Mbp) of *E. amylovora *is also the smallest among sequenced enterobacterial plant and mammalian pathogens [53]. However, *E. amylovora *still maintains both enterobacterial-specific TCSTs (such as the Rcs system) [54] and plant enterobacterial-specific ones (such as HrpXY) [39].

On the other hand, *E. amylovora *has almost the same number of TCSTs as that found in *E. tasmaniensis*, a saprophytic bacterium isolated from apple flowers in Tasmania, Australia [38]. Since both bacteria occupy the same ecological niche during colonization and can grow epiphytically on flowers, these bacteria might have developed and maintained similar TCSTs. This suggests that TCSTs may be evolutionarily maintained to cope with similar environmental and plant host signals in closely related bacteria. It is interesting to note that fire blight is endemic to North America, and it has subsequently spread to Europe and New Zealand in the 1950s and in 1917, respectively, but it has not yet been reported in Australia [14]. It is possible that these two bacteria may have not yet encountered each other, as the distribution of *E. tasmaniensis *outside of Australia remains unknown. Surprisingly, we found that the majority of TCSTs in these two bacteria share a high level of aa similarity and identity. These results suggest that similar TCSTs might play important roles for the survival and proliferation among closely-related bacteria in similar plant niches.

Currently, several thousands of TCSTs have been identified in sequenced bacterial genomes [55-57]. Although the basic biochemistry of TCSTs is well understood, some structural insights in phosphorylation-dependent changes of TCST domains are variable. Domain architecture has proven particularly informative for analyzing multidomain proteins involved in signal transduction and in predicting the functions of these signal transduction proteins [43,58]. In RRs, structural characterization of DNA-binding domains has revealed several variations on the common helix-turn-helix (HTH) theme, such as NarL- and OmpR-types. Recently, many novel conserved domains have been described such as PAS, GAF, GGDEF, EAL, and HD-GYP, thereby affirming the complexity of bacterial signaling systems [43,58]. In *E. amylovora*, most HKs belong to four common HK groups, and are periplasmic sensors. Moreover, most RRs in this bacterium are OmpR-, NarL-, NtrC-, and LytR-like proteins, the four most common families of DNA-binding RRs found in prokaryotes [43]. These results indicate that *E. amylovora *has maintained some basic signal transduction systems for the bacterium to survive. Phylogenetic and genomic analyses have revealed co-evolutionary relationships between cognate HKs and RRs. This seems obvious in *E. amylovora *as orphan HKs and RRs are rare.

It is well understood that TCSTs are involved in regulating virulence gene expression in plant bacterial pathogens [10]. Previous reports on TCSTs have demonstrated the importance of TCSTs in the virulence of bacterial plant pathogens. However, data on complete or global virulence regulation networks are lacking. A recent study by Qian *et al*. [13] provides a useful beginning towards a better understanding of the regulatory networks involved. A genome-wide mutagenesis of all 54 RRs in *X. campestris *pv. *campestris *has revealed that two novel RRs are involved in virulence, thus facilitating future studies on signaling networks in this bacterium [13]. In this study, we have utilized a reverse genetic approach and constructed 59 HK and RR mutants in *E. amylovora*, which will also provide valuable tools for future global gene expression assays using microarrays to deduce signaling networks in this bacterium.

Early studies have revealed that in *E. amylovora*, the Hrp T3SS, which delivers effector proteins into host plants, and the EPS amylovoran are two major virulence factors [52,59,60]. Previous reports have also indicated that the RcsCDB phosphorelay system regulates amylovoran biosynthesis, while the two-component system HrpXY regulates *hrp*-T3SS gene expression. Recently, we have further demonstrated that the Rcs system is essential for virulence in *E. amylovora *and may play a role in the survival of the pathogen [17]. Mutations in the Rcs system have rendered the organism non-pathogenic [17]. In this study, we have found that *hrpX*, *hrpY *and *hrpXY *mutants remain virulent, and could induce a spotty weak hypersensitive response (HR) on tobacco (Figure S2A, [see Additional file 2]); while, a *hrpXYS *triple mutant has a normal Hrp- phenotype. It is interesting to note that, in a previous report, Tn*5*-insertional mutants of *hrpY *have been reported to be non-pathogenic, and could not induce an HR on tobacco [18]. Two classes of *hrpX *insertional mutants have been identified, one similar to the *hrpY *mutant and the other that continue to cause disease and induce a spotty HR on tobacco [18]. Similar observations have been reported in *Pantoea stewartii *subsp. *stewartii*, causal agent of Stewart's wilt of corn [61]; wherein, Tn*5*-insertional *hrpX *and *hrpY *mutants exhibit a Hrp- phenotype and in-frame deletion *hrpX *mutants show reduced virulence. Since the *hrpXY *is transcribed as an operon, it is possible that Tn*5 *insertion could cause polar effects on the downstream genes such as *hrpS *(Figure S2B, [see Additional file 2]). Indeed, a recent study indicates that the *hrp *regulatory genes in *P. stewartii *subsp. *stewartii *participate in a novel regulatory loop that upregulates itself by readthrough transcription of *hrpL *into *hrpXYS *[62].

It has been proposed that in *E. amylovora*, both HrpY and HrpS regulate *hrpL*, encoding the master regulator of T3SS, and that the effects of HrpY and HrpS are additive [18]. Subsequent studies of *P. stewartii *subsp. *stewartii*, *D. dadantii*, and *Pantoea herbicola *pv. *gyposophilae *have demonstrated that HrpY initially activates *hrpS *by binding to its promoter, and then the HrpL is activated by HrpS as well as by other regulatory genes [39,63]. Furthermore, it has been reported that HrpY in *D. dadantii *acts as both a positive and negative regulator [39]. In *P. stewartii *subsp. *stewartii*, besides the HrpY binding site, additional sequences further upstream of the *hrpS *promoter are also required for *hrpS *expression, suggesting that unknown regulatory proteins may act cooperatively with HrpY [63]. Microarray studies suggest that *hrpL *represents only one branch of the regulatory pathways downstream of *hrpRS*, and a large number of genes regulated by HrpRS are *hrpL*-independent in *P. syringae *[64]. In our study, HrpX, HrpY, and HrpS also act as negative regulators, as *hrpX*, *hrpY*, *hrpXY*, and *hrpXYS *mutants produce more amylovoran. Our virulence tests suggest that the T3SS remains functional in *hrpX, hrpY*, and *hrpXY *deletion mutants, but not in the *hrpXYS *mutant, whereby HrpS and HrpL may be activated by other unknown regulators except that production and/or translocation of HrpN (Harpin) in tobacco is severely attenuated as showed in an HR assay (Figure S2A, [see Additional file 2]). It is possible that host signals affecting gene expression may also be different in tobacco.

Various models have proposed that HrpX senses environmental signals in the plant apoplast or the Hrp-inducing medium to phosphorylate HrpY [18,34,35]. However, domain structure analysis has indicated that HrpX is a soluble cytoplasmic protein, and may sense intracellular signals. This suggests that other signaling pathways may also be involved in activating *hrpXY*, *hrpS*, or *hrpL *by sensing outside signals to regulate T3SS. Since the expression of HrpX, HrpS, and HrpL is regulated by low pH, also corresponding to conditions under which OmpR-EnvZ and GrrS-GrrA are activated, our results further suggest that both OmpR-EnvZ and GrrS-GrrA can regulate the *hrpXY *operon or *hrpS *either directly or indirectly as reported in other plant pathogenic bacteria [10,28,33,34]. Further studies are needed to dissect the roles of OmpR-EnvZ and GrrS-GrrA in regulating T3SS.

Several regulatory genes have been previously reported to control amylovoran biosynthesis in *E. amylovora*, including the Rcs system, RcsA, Lon protease, and H-NS protein [17,50,65]. Here, we have further identified several groups of regulators, including both negative and positive regulators. These regulators may form a network that governs the production of amylovoran under different conditions to benefit pathogen survival or pathogenesis. Regulatory cascades are also likely to occur as global regulators such as OmpR-EnvZ and GrrS-GrrA may control expression of other regulatory genes or proteins, such as *hrpXY *and quorum sensing systems, as reflected in the amount of amylovoran produced in these mutants. However, we cannot rule out that cross-talk between different TCSTs may further complicate this scenario. Our study indicates that regulation of amylovoran biosynthesis is highly complex and further suggests that the pathogen has developed a system to control this major virulence factor.

Swarming is a flagella-driven form of motility for movement across solid surfaces as a group [66-70]. Swarmer cells are normally hyperflagellated and require extracellular components such as EPS and surfactants that enable mass migration [71,72]. Previous studies have identified several global regulators in *Es. coli *and in other bacteria, including the Rcs system, OmpR and GrrSA, known to influence flagella biosynthesis, especially the master regulator *flhDC *[71,73]. In this study, we have identified both negative (GrrSA) and positive (EnvZ/OmpR) regulators of swarming motility. It is easy to accept that the GacSA system may negatively regulate flagella biosynthesis, thus rendering *grrSA *mutants hypermotile. It is also obvious that flagella biosynthesis is not impaired in the *envZ*-*ompR *mutants as the swarming phenotype is different between the *envZ*-*ompR *mutants and the *flhDC*-*fliA *mutants. The obvious question that arises as to why *envZ*-*ompR *mutants are non-motile although they produce prolonged flagella. A recent study in *Salmonella typhimurium *has reported that mutations in chemotaxis pathways are impaired for swarming motility and further revealed a role of flagellum in sensing external wetness [72]. It has been proposed that swarming requires a fluid environment generated as bacteria extract water from the underlying agar gel [74]. Flagella are designed to work in this aqueous environment; that is swarming cells move in a thin layer of fluid over the surface of the agar. Further studies have revealed that the wetting agent that draws water out of the underlying agar is an osmotic agent [74]. It is likely that in our *envZ*-*ompR *mutants, this unknown osmotic agent provides a signal for EnvZ-OmpR system that may regulate chemotaxis response or creates high osmolarity so that water can be removed from the agar, thus affecting swarming motility. Further studies are needed to clarify this hypothesis.

## Conclusion

In summary, we have identified and classified TCSTs, and have systematically generated TCST deletion mutants in *E. amylovora*. The mutants generated will serve as resources for future exploration of TCSTs in *E. amylovora*. In-depth characterization of deletion mutants and global gene expression will be our next goal. Our current data provide experimental evidence that TCSTs, especially those global regulators such as the Rcs system, EnvZ-OmpR and GrrA-GrrS, play important roles in virulence and in regulating virulence factors such as amylovoran production and swarming motility. These findings also suggest that multiple TCSTs may form complex and highly connected circuits and signaling networks in this important pathogen.

## Methods

### Bacterial stains and culture media

Bacterial strains and plasmids used in this study are listed in Table 4. The LB medium was used routinely for culturing *E. amylovora*. When necessary, the following antibiotics were added to the medium: 20 μg ml^-1^kanamycin and chloramphenical, and 100 μg ml^-1 ^ampicillin. Amylovoran production was determined by growing bacteria in MBMA medium (3 g KH_2_PO_4_, 7 g K2HPO_4_, 1 g [NH_4_]_2_SO_4_, 2 ml glycerol, 0.5 g citric acid, 0.03 g MgSO_4_) amended with 1% sorbitol [52,75].

**Table 4:** Bacterial strains and plasmids used in this study

Strains or plasmids	Relevant characters^a^	Reference or source
*E. amylovora *strains		
Ea1189	Wild type, isolated from apple	81
Z2946*ΔflhD*	*flhD::Km; *Km^r^-insertional mutant of *flhD *of Ea1189, Km^r^	This study
Z2945*ΔflhC*	*flhC::Km; *Km^r^-insertional mutant of *flhC *of Ea1189, Km^r^	This study
Z2986*ΔfliA*	*fliA::Km; *Km^r^-insertional mutant of *fliA *of Ea1189, Km^r^	This study
Z0118*Δams*	Km^R^-deletion mutant of *ams *operon (15.8 kb) of Ea1189, Km^R^	47
*E. coli*		
DH10B	F^- ^*mcr*A Δ(*mrr*-*hsd*RMS-*mcr*BC) Φ80*lac*ZΔM15 Δ*lac*X74 *rec*A1 *end*A1 *ara*Δ139 Δ(*ara*, *leu*)7697 *gal*U *gal*K λ – *rps*L (Str^R^) *nup*G	Invitrogen, Carlsbad, CA, USA
Plasmids		
pKD46	Ap^r^, P_BAD _gam bet exo pSC101 oriTS	45
pKD13	Km^r^, FRT cat FRT PS1 PS2 oriR6K rgbN	45
pKD3	Cm^R^, FRT cat FRT PS1 PS2 oriR6K rgbN	45
pGEM^® ^T-easy	Ap^r^, PCR cloning vector	Promega, Madison, WI, USA
pWSK29	Ap^r^, cloning vector, low copy number	82
pFPV25	Ap^r^, GFP based promoter trap vector with a promoterless *gfpmut3a *gene	83
pWDP4	721 bp KpnI-XbaI DNA fragment containing promoter sequence of *amsG *gene in pFPV25	17
pWDP5	A 3.057 kb PCR fragment containing *grrS *gene in pGEM T-easy vector	This study
pSN4	A 2.727 kb PCR fragment containing *envZ/ompR *operon in pGEM T-easy vector	This study
pSN5	A 2.727 kb PCR fragment containing *envZ/ompR *operon in pWSK29 vector	This study
pSN6	A 1.6 kb PCR fragment containing *grrA *gene in pGEM T-easy vector	This study
pSN7	A 1.463 kb PCR fragment containing *flhDC *operon in pWSK29 vector	This study
pSN8	A 940 bp PCR fragment containing *fliA *gene in pWSK29 vector	This study

^a ^Km^R^, Ap^R^, Cm^R ^= kanamycin, ampicillin and chloramphenical resistance, respectively.

### DNA manipulation and bioinformatic analysis

Plasmid DNA purification, PCR amplification of genes, isolation of fragments from agarose gels, cloning, restriction enzyme digestion and T4 DNA ligation were performed using standard molecular procedures [76]. DNA sequencing was performed at the Keck Center for Functional and Comparative Genomics at the University of Illinois at Urbana-Champaign. Sequence management and contig assembly were conducted using Sequencher 4.9 software. Similarity searches of nucleic acid sequences and proteins were conducted using the BLASTN and BLASTP programs at NCBI [77]. Protein domain organizations of the HKs and RRs were identified by searching the CDD with Reverse Specific Position BLAST [41] or the SMART program [42]. Domain limits for proteins were also derived from the graphical output of the SMART web interface. Transmembrane segments of HKs were predicted by the *TMHMM2 *program [78]. Multiple sequence alignments of proteins were carried out using Clustal ×1.80 [79]. Sequence logos were derived from alignment of proteins belonging to corresponding HK group and created by Weblogo . Proteins without known domain were characterized by using PSI-BLAST of NCBI and methods described previously [80].

### Construction of TCST mutants in *E. amylovora *by Lambda-Red recombinase

*E. amylovora *stable mutants were generated by using the λ phage recombinases as previously described [45-47]. Briefly, *E. amylovora *Ea1189 was transformed with plasmid pKD46 expressing recombinases redα, β, and γ. The transformant Ea1189 (pKD46) was grown overnight at 28°C, reinoculated in LB broth containing 0.1% arabinose, and grown to exponential phase OD_600_= 0.8. Cells were collected, made electrocomponent, and stored at -80°C. Recombination fragments consisting of a kanamycin (*kan*) or chloramphenical (*cat*) gene with its own promoter, flanked by a 50-nucleotide (nt) homology arm, were generated by PCR using plasmid pKD13 or pKD3 as a template. The primers used for generating mutants are listed in Table S2 [see Additional file 1] (primers start with B). To confirm mutants by PCR, primers (Table S2, [see Additional file 1], primers start with Z), and internal primer pairs km1 and km2 of the *kan *gene, cm1 and cm2 of the *cat *gene were used. For the resulting mutants, the majority of the coding region of each gene was replaced by the *kan *or *cat *gene, except for the first and last 50 nt. The resulting mutants were designated and listed in Table 1 and Table 3.

### Cloning genes/operon for complementation of TCST mutants

For complementation of selected mutants, flanking sequences of genes or operon were used to design primers to amplify fragments of gene/or operon and their promoter sequences (Table S2, [see Additional file 1], primers with gene name). Primer pairs, with or without restriction sites were used to amplify corresponding DNA fragments from *E. amylovora *WT strain which contains upstream and downstream sequences of the gene or operon, and PCR fragments were cloned into either pGEM T-easy vector through A-T ligation or into a low-copy number vector pWSK29. The final plasmids were listed in Table 4. For primer pair with restriction site, DNA fragments and the vector were both digested with corresponding enzymes following amplification, and ligated together. All plasmids were introduced into *E. amylovora *strain by electroporation. Transformants were selected on LB plates supplemented with Ap and Km or Cm. Their genotypes were confirmed by both enzymatic digestion and sequencing.

### Construction of promoter-GFP fusions for gene expression assays

For gene expression assays, flanking sequences of the *amsG *ORF were used to design primers to amplify DNA fragments. Primer pairs amsG1-amsG2 with restriction sites were used to amplify 721 bp DNA fragments from *E. amylovora *WT strain, containing promoter sequences of *amsG *gene. PCR products and the promoter trapping vector pFPV25 were both digested with *BamH*1 and *EcoR*I for *amsG *gene. The resulting fragments were gel-purified, ligated together, and cloned to the upstream of promoter-less *gfp *gene. The final plasmids were designated as pWDP4 for *amsG*, and were confirmed by restriction enzyme digestion and sequencing.

### Immature pear virulence assays

For *E. amylovora *WT and mutant strains, bacterial suspensions were grown overnight in LB broth, harvested by centrifugation, and resuspended in 0.5 × sterile phosphate buffered-saline (PBS) with bacterial cells adjusted to concentrations of ~1 × 10^3 ^to 1 × 10^4 ^colony-forming units (CFU/μl) (OD_600 _= 0.1 and then diluted 100 times) in PBS. Immature fruits of pear (*Pyrus communis *L. cv. 'Bartlett') were surface-sterilized, and pricked with a sterile needle as described previously [19,46]. Wounded fruits were inoculated with 2 μl of cell suspensions, and incubated in a humidified chamber at 26°C. Symptoms were recorded at 2, 4, 6, and 8 days post-inoculation.

### CPC assay for determining amylovoran concentration

The amylovoran concentration in supernatants of bacterial cultures was quantitatively determined by a turbidity assay with cetylpyrimidinium chloride (CPC), as previously described [50,52]. Briefly, for *E. amylovora *WT, mutants, and complementation strains, bacterial suspensions were grown overnight in LB broth w/o appropriate antibiotics, harvested by centrifugation, and washed with PBS three times. After the final wash, the bacterial pellet was resuspended in 200 μl PBS. A total of 100 μl of bacterial suspension was inoculated into 10 ml MBMA medium with 1% sorbitol. One ml of bacterial cells was pelleted two to three days after inoculation at 28°C with shaking. Following centrifugation, 50 μl CPC at 50 mg ml^-1 ^was added to one ml supernatant. After 10 min of incubation at room temperature, the amylovoran concentration was determined by measuring OD_600 _turbidity. The final concentration of amylovoran production was normalized for a cell density of 1.0. For each strain tested, the experiment was repeated at least three times.

### Swarming motility assay

For *E. amylovora *WT and mutant strains, bacterial suspensions were grown overnight in LB broth w/o appropriate antibiotics, harvested by centrifugation, washed with PBS once, and resuspended in 200 μl PBS. Then, bacterial suspensions were diluted 10 × in water, and 5 μl of the diluted bacterial suspension was plated onto the center of swarming agar plates (10 g tryptone, 5 g NaCl, 3 g agar per l Liter) as previously described [50,51]. Swarming diameters were determined following incubation at 28°C for up to three days. The experiments were repeated at least three times.

### Statistical analysis

Amylovoran production and swarming motility data were undertaken a one-way ANOVA and Student-Newman-Keuls test to determine differences in means (P = 0.05) using SAS 9.1 program.

### GFP reporter gene assay by flow cytometry

The BD FACSCanto flow cytometer was used to monitor the GFP intensity of WT and mutant strains containing the corresponding promoter-*gfp *construct [17]. For *in vitro amsG *gene expression, WT and mutants containing the *amsG *promoter-GFP fusion plasmid were grown in LB overnight, harvested, and resuspended in water. Bacterial suspensions were re-inoculated in MBMA broth with 1% sorbitol and grown at 28°C with shaking for up to three days. Bacterial cultures were then harvested by centrifugation, washed once with PBS, and then resuspended in PBS for flow cytometry assay. Flow cytometry was performed on a BD LSRII 10 parameter multilaser analyzers (BD Bioscience, San Jose, CA). For both cases, data were collected for a total of 100,000 events and statistically analyzed by gating using flow cytometry software FCS Express V3 (De Novo Software, LA, CA). A geometric mean was calculated for each sample. Each treatment was performed in triplicate and each experiment was repeated three times.

## Abbreviations

CDD: Conserved Domain Databases; CPC: cetylpyrimidinium chloride; EAL: Type I c-di-GMP phosphodiesterase; EPS: extracellular polysaccharide; HisKA: histidine kinase domain; HK: histidine kinase; HMM: Hidden Markov Model; Hpt: histidine phosphotransfer; HTH: helix-turn-helix; GGDEF: diguanylate cyclase; PAS: initially found in *P*ER, *A*RNT, and *S*IM proteins; REC: receiver domain; RR: response regulator; TCST: two-component signal transduction system; T3SS: type III secretion system.

## Authors' contributions

YFZ designed and coordinated the project, YFZ, DPW, and SN performed the experiments and analyzed the data; GWS and SSK contributed reagents/materials, analyzed and discussed the results; YFZ wrote the manuscript and GWS and SSK critically read the manuscript. All authors have read and approved the final manuscript.

## Supplementary Material

Additional File 1 **Comparison and classification of TCSTs in *E. amylovora *and other related enterobacteria and primers used in this study**. The file contains two tables. Table S1 compares and classifies TCSTs in *E. amylovora *and other related enterobacteria; Table S2 lists primers used in this study. Click here for file

Additional File 2 **Structural classification of histidine kinases, hypersensitive response of HrpXYS mutants in tobacco and schematic map of HrpXYS operon**. The file contains two figures. Figure S1 shows structural classification of histidine kinases (HKs) based on phosphorylation sites (H-boxes); Figure S2 shows hypersensitive responses (HR) on tobacco for WT, ΔT3SS [47], *hrpX*, *hrpY*, *hrpXY*, and *hrpXYS *mutants (Figure S2A) and a schematic map of the HrpXYS operons (Figure S2B). Click here for file
