# Supplementary material for: Systems level analysis of two-component signal transduction systems in Erwinia amylovora: Role in virulence, regulation of amylovoran biosynthesis and swarming motility
Source: BMC Genomics. 2009 May 26;10:245. doi: 10.1186/1471-2164-10-245 (PMC2698875; doi:10.1186/1471-2164-10-245)
Supplement: Additional File 1 — Comparison and classification of TCSTs in E. amylovora and other related enterobacteria and primers used in this study. The file contains two tables. Table S1 compares and classifies TCSTs in E. amylovora and other related enterobacteria; Table S2 lists primers used in this study. [file 1471-2164-10-245-S1.pdf]

Table S1. Comparison and classification of TCSTs in *E. amylovora* and other related enterobacteria

E. amylovora Ea273	*P. carotovora* SCRI1043 [a]	*D. dadantii* 3937[a]	*E. coli* K12 [b]	HK type [c]	RR type [d]
**Metabolic responses**					
ArcBA	Eca0314-3893	+	+	IB hybrid	OmpR-like
DcuSR	Eca4398-4399	+	+	II	OmpR-like
NarQP	Eca1900-1901	--	+	III	NarL-like
NtrCB	Eca0027-0028	+	+	IC	NtrC-like
**Ion/metal responses**					
BaeSR*	Eca3188-3189	+	+	IA	OmpR-like
KdpDE	Eca1338-1339	+	+	IA	OmpR-like
PhoQP	Eca2445-2446	+	+	IA	OmpR-like
PhoRB	Eca1110-1111	+	+	IA	OmpR-like
PmrBA (BasSR)	Eca4044-4045	+	+	IA	OmpR-like
**Stress responses**					
EnvZ/OmpR	Eca4107-4108	+	+	IA	OmpR-like
CpxA1R1	Eca4311-4312	+	+	IA	OmpR-like
CpxA2R2	Eca0889-0890	+	--	IA	OmpR-like
GrrSA	Eca2882-3571	+	+	IB hybrid	NarL-like
RcsCDB	Eca1201-03-04	+	+	IB hybrid; HPT	NarL-like
**Unknown**					
RstBA	Eca2012-2013	+	+	IA	OmpR-like
YfhKA	Eca3255-3257	+	+	IA	NtrC_like
YehUT	Eca3353-4152	+	+	IV	LytR-like
**T3SS**					
HrpXY	Eca2088-2089	+	--	III	NarL-like
**TCSTs not present in *E. amylovora* but in other related enterobacteria**					
CitAB	Eca2577-2578	+	+	II	NA
NarXL	Eca2028-2029	+	+	III	NarL-like
UhpBA	Eca1496-1497	+	+	III	NarL-like
YpdAB	Eca2431-2432	+	+	NA	NA
CusRS	--	+	+	IA	OmpR-like
QseCB	Eca0007-0008	--	+	NA	NA
One HK/RR	Eca0785-0786	--	--	NA	NA
7 HKs/RRs [a]	--	+	--	NA	NA
5 HKs/RRs [b]	--	--	+	NA	NA

[a] Data for *Dickeya dadantii* (*Erwinia chrysanthemi*) 3937 and *Pectobacterium* (*Erwinia*) *carotovora* subsp. *atroseptica* SCRI1043 see Yap *et al*., 2008. Seven HKs and RRs only in *D. dadantii* are TctE/D; ABF-0016854/16853; 19562/46568/14657; 15830/15831; 15809/15810; 16068/16069; 14915/14914. Two sets of CheABRWYZ exist in *E. amylovora*, but only one set in other related enterobacteria. +: present; ---: absent.

[b] Data for *E. coli* K12 see Kim and Forst, 2001. Five HKs and RRs only in *E. coli* are AtoS/C; TorS/R; CopS/R; EvgS/A and CreC/B. +: present; ---: absent.

[cd] HK families see Kim and Forst, 2001. RRs see Galperin, 2006. NA: not analyzed.

Table S2. Primers used in this study

Primer	Sequences (5'—3') [a]
B0089F	GTGTCCCCCTGGTGCGCTTCGAAAGTCGCCTGTATAAGGACGTTTACCGG GCGATTGTGTAGGCTGGAGCT
B0089R	TCAGCGCTCTTCCAGCCGGTAACCCACGCCGCGTACCGTTTTAATCCGAT ATTCCGGGGATCCGTCGACC
B0090F	ATGAACAGTATGCGCAGGCGTTTGCTGGTGATGCTGGCGCTGATCGTGCT GCGATTGTGTAGGCTGGAGCT
B0090R	CTAACCCAGCTGCTTTGGCAGCCAGCAGCAGACATCCAGGCCACAGCAAT ATTCCGGGGATCCGTCGACC
B0117F	ATGAAGCAAATTCGTTTGTTAGCGCAGTACTACGTTGATTTAATGGTAAA GCGATTGTGTAGGCTGGAGCT
B0117R	TCATTTTTTTCAGCGTCTGTTACCCATTGCCGTAAAACTTCCATATCAT ATTCCGGGGATCCGTCGACC
B0270F	ATGAGGCGGCTCCGCTTCTCTCCCCGTAGTTCATTTGCGCGTACGCTGTT GCGATTGTGTAGGCTGGAGCT
B0270R	TTATGCCACCGGGCCGGCCGGCAAGCTGGCTGGCGGCAGCGGCAGATAAG ATTCCGGGGATCCGTCGACC
B0271F	ATGAAAGATAAGCTGCTGTTTAATATGCTTTGTAACAATTTCGGCTACAA GCGATTGTGTAGGCTGGAGCT
B0271R	TCATGCTTTGCTGCCGTCCGGGACAAAGACATAACCCAAACCCCAGACGG ATTCCGGGGATCCGTCGACC
B0355F	TTGCAAGCATTTGTAAAGCCGAAGCATGAACGTATCTGGCTGGTGGCATC GCGATTGTGTAGGCTGGAGCT
B0355R	CTAAGCATTCTGCTGATCTTTCCACTGCATCAGCGCAGCGCTCGACATGG ATTCCGGGGATCCGTCGACC
B0414F	ATGAACGACGAACTCCAGCGCCCCAATCCGGATGCGCTGCTACGCCAGGC GCGATTGTGTAGGCTGGAGCT
B0414R	CTACATTGGCGTGTAATCTTCCAACAGCGGTGGTGCCGTTAGCGGCAGAG ATTCCGGGGATCCGTCGACC
B0686F	ATGATATCCAGCCTGACCACCCGTATCTTTGCCATCTTCTGGCTTACTTT GCGATTGTGTAGGCTGGAGCT
B0686R	TTATTCGCTGTGAGTGGTATACAGCGGCAGCCATATTGTCAGGCGCAAAC ATTCCGGGGATCCGTCGACC
B0687F	ATGAATAAGATCTTATTGGTTGACGACGACCGCGAATTGACTTCGCTACT GCGATTGTGTAGGCTGGAGCT
B0687R	TCATGTTGCCGATACCATCAGGTATCCCCGGCCTCGCAAGGTTTTAAACC ATTCCGGGGATCCGTCGACC
B0955F	ATGAATGAAGACTCAGATGTTGTGTACCAGCTTTTGGCAAGAAGCGAGGG GCGATTGTGTAGGCTGGAGCT
B0955R	CTAAAATTCTTTGAGGCGGCGCTCCGGCGGCTGCGGGCGGCCAAAATAAT ATTCCGGGGATCCGTCGACC
B1215F	ATGAATTTGCAAAGCTACGATGAGTTACTGGACAGCAAGCAACGGCTGTC GCGATTGTGTAGGCTGGAGCT
B1215R	TTATACGATATCTGCAGGTTTACCGAGCGGCATTTTTGCCGCGTCCTGAA ATTCCGGGGATCCGTCGACC
B1250F	ATGTCACATCAGGATAACAGCCAGGGGATGCCGAATGCATTACCGGTTGG GCGATTGTGTAGGCTGGAGCT
B1250R	TTAGCTTTGCGGATTTAACAACCACGCACCTTCATGTTCGATGGTGATGG ATTCCGGGGATCCGTCGACC
B1306F	GTGACCTCCATATTGGGTGCCAGGCCGTTGTTGAAGGTACAGGGATGCCG GCGATTGTGTAGGCTGGAGCT
B1306R	TCAGTCCTGTAATTCACCACAGAAACGGTAACCTTCGCCGTGAATGGTGG ATTCCGGGGATCCGTCGACC

B1675F	ATGGCAAAGCGCATTCTTGTGGTCGAAGATGAAGCCCCAATCCGTGAGAT GCGATTGTGTAGGCTGGAGCT
B1675R	TCAATAACGTGCAGAGAAACGATAACCTGTTCCGCGAACCGTCTGCACCA ATTCCGGGGATCCGTCGACC
B1676F	GTGTTGGAACGCCTCTCCTGGAAGAGATTACTGTTTGAGCTGCTGCTTGC GCGATTGTGTAGGCTGGAGCT
B1676R	TTAAGGCCGCATGCAGCCGCCCGGTTCAGCACCCACAATCATCCTGAGCG ATTCCGGGGATCCGTCGACC
B1912F	TTGACCGACCGGCGAGGTATTGTGACCACCATTTTGATTGTTGAAGATGA GCGATTGTGTAGGCTGGAGCT
B1912R	TTATGGCATAAAGCGATATCCAACGCCGGTTTCGGTGAGAAGATGCTGCG ATTCCGGGGATCCGTCGACC
B2074F	ATGCATATGGTTTCACTGGTCGTCCCGGATCTCGACGTGCTACGACGTTG GCGATTGTGTAGGCTGGAGCT
B2074R	TTAATGCAGCAGGGAATGGCTGGTTATCATTGGAGGTCGTTCTTCATCTT ATTCCGGGGATCCGTCGACC
B2194F	TTGATGACATCTGAAAACTATTTCATTTGGCGAGCAGATATCAAATCAGC GCGATTGTGTAGGCTGGAGCT
B2194R	TCAGATTAAACCGATTGCTGCTGCATAGGAGGCAATTTGAGTTTTGTTAG ATTCCGGGGATCCGTCGACC
B2195F	TTGCAAGGTGGTTGTCTAACCTGCGCCCAAAGTCATACAGCTGCTCAAAC GCGATTGTGTAGGCTGGAGCT
B2195R	TTAATCTTTATGGCGTCCACGCAGCCCAAAATTGGCGGAGACGCCGAATG ATTCCGGGGATCCGTCGACC
B2198F	TTGATTAGCGTTTTTCTTGTTGATGACCATGAGCTGGTGCGCGCAGGTAT GCGATTGTGTAGGCTGGAGCT
B2198R	TTACTCACTACTGATTAATGACTCCGCACTGAACAGACCATGTCGAATGG ATTCCGGGGATCCGTCGACC
B2323F	ATGTGTAATTCACGTTTTCGCCGTATAAAACCCTTTTCGTTACGCATCCG GCGATTGTGTAGGCTGGAGCT
B2323R	TTACTGGCGCTGAAAAATGACTTCCATTCGTGCGCCGCCAAGCGTGCTGG ATTCCGGGGATCCGTCGACC
B2324F	ATGCGTGTGTTAGTTGTGGAAGATAATCCACTGCTGCGTCATCATTTGGC GCGATTGTGTAGGCTGGAGCT
B2324R	TCAGAGATCGAAGCGGTAACCCTGACCGCGCACCGTAGTGATCACTTCCT ATTCCGGGGATCCGTCGACC
B2361F	ATGATCAATGTTTTAATCGTCGATGATGATGCGATGGTGGCAGAACTTAA GCGATTGTGTAGGCTGGAGCT
B2361R	TCACTGGCAGAACCGCTTAAGCAGAATGCTGCGTTCTGCCTGAAGACGGT ATTCCGGGGATCCGTCGACC
B2362F	TTGCCTGCCCCATTGCGCGCTACAGGAGTCAATATTGAAACGGCTCAGCC GCGATTGTGTAGGCTGGAGCT
B2362R	TCATGCTTTTTTGAGCTCTCCATCCCAGGGCAGTTGTACAAAAAATTGCG ATTCCGGGGATCCGTCGACC
B2651F	TTGCTAACCCATATATTTGGTTCTATGAACAAAATTGTATTCGTTGAAGA GCGATTGTGTAGGCTGGAGCT
B2651R	TCATGACGCATCCCAGGCATGAGGGGCAAACAGGTAGCCCTTGTTTCTGA ATTCCGGGGATCCGTCGACC
B2662F	ATGAGGAAGCTGTTTGTCCAGTTTTACCTGCTGCTGTTCGTTTGCTTCCT GCGATTGTGTAGGCTGGAGCT
B2662R	TTAGTTGAGCGCAGCAGGCCAGCAAAAACGTATGCTGGCACCGCCCAGTG ATTCCGGGGATCCGTCGACC
B2946F	ATGGGTACATCAGAATTACTCAAACAAATTTACGACATCAATTTGTCTTA GCGATTGTGTAGGCTGGAGCT
B2946R	TTAGCTGGCTGCCCTTTTTTTCGGCGTCTCTTGTTCTTTCGTCACGTTAC ATTCCGGGGATCCGTCGACC

B2986F	GTGAACGATCTGTATACCGCCAATGGCGTGATGGACAAACATTCGCTGTG GCGATTGTGTAGGCTGGAGCT
B2986R	TCAGCGCGCTCCCGATAACCTGGCGCGCAGGCGTTTTATCGCCTGACTGT ATTCCGGGGATCCGTCGACC
B3113F	ATGTCGACAGATAATTCAAGACCGTTGCTGCAACTGCGCTCGCTCTGTCT GCGATTGTGTAGGCTGGAGCT
B3113R	TCAGTACACGGAAAGCCGACCGCGCCCGCTGTGTTTGGCGTGATAACAGG ATTCCGGGGATCCGTCGACC
B3119F	ATGTTGGCAAAAATCCGTATCGGCATTACCGCCAAGCTGTTCGCCGCCAT GCGATTGTGTAGGCTGGAGCT
B3119R	TCAATGTGCCTGATACGGTAAGTGTATCGTAATTCTCACGCCTCCGGCAG ATTCCGGGGATCCGTCGACC
B3120F	TTGACCCGGGCTGACGAAATGAACCAGATCCCCGCCACTCCACTGATTTT GCGATTGTGTAGGCTGGAGCT
B3120R	CTAGATCAGACGACAGCCATCTGCTTCCCAGCGATAGCCGATGCCGTACA ATTCCGGGGATCCGTCGACC
B3205F	ATGTTGCCATATAAATTTCCGCTAACTTCCGGCAATGTAACACGATTTTT GCGATTGTGTAGGCTGGAGCT
B3205R	TTATTGGTTACCTTGCTGCAGGAGATCTTTGACATAAGTGTCAACTTCAC ATTCCGGGGATCCGTCGACC
B3206F	GTGAAGTTGACACTTATGTCAAAGATCTCCTGCAGCAAGGTAACCAATAA GCGATTGTGTAGGCTGGAGCT
B3206R	TTATTTATCTACCGGCGTCATGCTTACTGATGACAGGTAGTTAAGCAAAG ATTCCGGGGATCCGTCGACC
B3207F	ATGGCCGGGATAACTGGCTTTTTCTGCCTCCCGGCGCTACTGTATCGGCA GCGATTGTGTAGGCTGGAGCT
B3207R	TTACCGCAACCTGTTACTTAGCGCTTCCCGGCTCTTTCTTACCCGCCCGG ATTCCGGGGATCCGTCGACC
B3324F	TTGACGACGATGATGGACAAAAAAACAACAAACCTGGAAAAAGTCCACGA GCGATTGTGTAGGCTGGAGCT
B3324R	TCAGTCTTTGAAAAATAGGTTATTCAAGAGAATGGAAGAGATAATAAAGG ATTCCGGGGATCCGTCGACC
B3327F	ATGAAAAGAACCTATCTGTATGTCGTTATCCTTGCACTTGTTTTTACAGT GCGATTGTGTAGGCTGGAGCT
B3327R	CTATGCAGGTTCTACAGCAACGCGCACCAGGAATGTTGACCCCATATTCA ATTCCGGGGATCCGTCGACC
B3367F	ATGAAATCGGGCTTACGTGGCTTCCTGTTCTGGAAGATCCTGCTGGGATT GCGATTGTGTAGGCTGGAGCT
B3367R	TCAACCGCCCGACTCAATCTCCCAGTGCGGCAGACGAATTGTCAGCTCCA ATTCCGGGGATCCGTCGACC
B3368F	TTGTTATACTGTAAGGATTGGGTAAAAGCGGCTGCGCATTATCCATTAAC GCGATTGTGTAGGCTGGAGCT
B3368R	TCATCGGATCCGGTAACCGATACTGCGCACCGTTTCGATAGCAATGGTGT ATTCCGGGGATCCGTCGACC
B3389F	ATGATCATGACCAGGAAATATGCCAATGTCAGGCAGGCTAACCGTCGTCT GCGATTGTGTAGGCTGGAGCT
B3389R	TTAAATGACCTCGGTCATCGCGGTAAAGTTCAGAGATACGCAGGTACCTC ATTCCGGGGATCCGTCGACC
B3390F	ATGGAAGCACAAAAGTACAGCGTATTAATTGTGGACGATCATCCATTGTT GCGATTGTGTAGGCTGGAGCT
B3390R	TTAGCGACAGAATTCCAGCCAGAGTACGGTTGCCGCCACACGCGATCTGA ATTCCGGGGATCCGTCGACC
B3495F	ATGATCAAAGCATCAGCGCGACTGCTGCTGGTCGATGATGACCCCAGTTT GCGATTGTGTAGGCTGGAGCT
B3495R	TTATCCTTTGAAATCGGCCGCATCGAGTTCATGGCGAGACAACAGTTTAT ATTCCGGGGATCCGTCGACC

B3498F	GTGAAGAAATGGCGTCTGTTTCCCTGTTCCCTGCGCCAGCTGGTGCTGATGCGATTGTGTAGGCTGGAGCT
B3498R	TTAGCTGCATGATTCTCCTTTGCCGGCGGGAGTATTAAATTCAATACGGAATTCCGGGGATCCGTCGACC
B3742F	ATGACCAAATACAGCCTGCGGGCACGCATGATGATTTTGATTCTGGCACCGCGATTGTGTAGGCTGGAGCT
B3742R	TTACAGCTTCAGCCGCTCTTTGGCCAGTCGTGCCACGTTATGCATTTCGTCCATGGTCCATATGAATATCCTCC
B3962F	ATGAACATCAGGAATAGTGAACACTCGTCCCGGCCATGGCCGGGACGGAGGCGATTGTGTAGGCTGGAGCT
B3962R	CTACTGAGCAATAACCCGACCGGTGTTCACGTTCAATAATTTGATGCGGTATTCCGGGGATCCGTCGACC
B3963F	ATGGATAAAAATATTCGCCTGATGGTAGCGGACGACCATGTCATCATGCGGCGATTGTGTAGGCTGGAGCT
B3963R	CTAAGTTATCAGGCAATGACTAATGGCGTATTTCACGATGTCCGCATTGGATTCCGGGGATCCGTCGACC
B3964F	ATGCAATTTTCCGAGCGACAGGTGAGCGCGAGCAGTGAGCCGCTCAAATTGCGATTGTGTAGGCTGGAGCT
B3964R	TTATTCCATAATCTGCTTATTTTCATAAGCTTGTACAATAGGAATGGTTAATTCCGGGGATCCGTCGACC
B3995F	ATGTTCAGCGTATTGATTGTCGATGACGAGCCGCTGGCCCGCGACAATCTGCGATTGTGTAGGCTGGAGCT
B3995R	TTATAGCCCGATGGCCTCCTTCAGGCTTTTCAGGTAGCGACGGCTCACCGATTCCGGGGATCCGTCGACC
B3996F	ATGTACGAATTGAACTCAATTTTACTGCTGTTACAGCAGATGTGTGTGTTGCGATTGTGTAGGCTGGAGCT
B3996R	TCAATACGCTGAACATACTGGCTCCAGCGGCAGACGCAGAGTAATGCGGGATTCCGGGGATCCGTCGACC
Z117F	GCCTGTCCCCGCATCTCTACCTGT
Z117R	ATGCTCAAATTCGGGTCTCCTGTT
Z1215F	CGGCTGTCGTTGATGCTGTTTCT
Z1215R	TGAGACGCACGGCATGAAGATTT
Z1250F	CAGCAGGGCGAATTCCAGTGTC
Z1250R	CGGCAGCTGCACGGTGATACGAT
Z1306F	GCTGCGCGGTGCCCAACA
Z1306R	CCGTGCCGATTATGCCGTGGAG
Z1675F	TGTCACCGCAGCAGGAGCAATAA
Z1676R	GCCATCAGGTTGCCAAGCGTATC
Z1912F	GTAATCAGGCCAAATCCGCAACAA
Z1912R	TGCCGATCACCGCGAGAAAATAG
Z2074F	GAAAGTCAGCGCAGCACAGGATG
Z2074R	CTGGAAAGCCCCCGGACTAAAA
Z2194F	CGGGCGTGCACAGGTCGTA
Z2195R	GGCGGCAGTAGTGGTGTTGATAGC
Z2198F	TTACCCGTTATTTGCAGTTGTTCCG
Z2198R	TGGGTTACCGTCACGTCTATCTGC
Z2323F	CGTACTCAGGAAGTGCGTCA
Z2323R	ACCCTTTGTAACGCGCTTGCTGTC
Z2324F	AAGCAAATGACGCCGGCAAACTAC
Z2324R	CCATTTAGCCAGGGTGAAAA

Z2361R	TGCGAAAGGGCCTGGAAACAC
Z2362F	ACACTGACCTGGCCCCACCTACTT
Z2651F	CTGACAGCCGCGATGCCGTAAC
Z2651R	GCTGTGGGTGGAAAATGGCTAAAA
Z2662F	GTACCAGACGCGCCACACCTATG
Z2662R	GCGAGCCGGCAATGGACTTAGC
Z270R	ATCTGGCCCGGCGTTTTATCA
Z271F	CGTGGTTATGCCGCTATTGTGTTG
Z2946F	CAGCAACCAGGTAGCGGTAT
Z2946R	TTGCCAGGCATTACAGAACA
Z2986F	CCGCTATTCCCTCAATCAAA
Z2986R	TAAGGTTCCCGGTAAAGTTGAA
Z3113F	CAGATTGCCGGCATTGAACACG
Z3113R	GCAGGCCACGCAAGCAACAGC
Z3119F	CGCTGCCCACCACGCCCAATG
Z3120R	GCAGCGGCCGGAGAAAGCAGTG
Z3205F	GCCAACGCCCGACTGATTACAAG
Z3206R	GCCGGGAAGCGCTAAGTAACAGGT
Z3207F	GTTCGCCGCTAAGCCGTGGTCA
Z3207R	GAGAAAATCAGCGCGGGTGGTTAC
Z3324F	CTGTCCTTTTGCTGTCTGGCTTCC
Z3324R	CCGCCACCTTGATTGATGTCTTCC
Z3327F	AACCAACTTCACGCCACAGAGCAT
Z3327R	GGAGGGCGGCGAACTGTGAA
Z3367R	CGTTGCCGGCGCCTGACCTGTA
Z3368F	TGTGACCGTGGTGACCGCTACTTA
Z3389F	GCCCCGGACGCTTCTTACCA
Z3390R	GCCCCTGTCCCTCGCTCACCATC
Z3495R	TCTGGCGACCGAATCCTTTGACTT
Z3498F	CGGACAAGCGCCCATAACATTC
Z355F	CGCTGCGGTCTGTTCGTTCG
Z355R	ACCGTGCACGCGAGTAAAACCATC
Z3742F	CGTTATTGTCTGGCGGGTCGTCAC
Z3742R	CGTTACAGGAAGCAGCGGAGAATG
Z3962F	TCCCTACATTGCCCCCTGAAGATT
Z3962R	TGATAACCGGCGACAGCACATTC
Z3963R	AGGAATCTTCAGGGGGCAATGTAG
Z3964F	TGCCGTCTGGACACTGCCTCAA
Z3995R	CCTTTGCTGGCGGTGTTTTGCTAC
Z3996F	GATCGCCGCTGTCAGGGTATGTTG
Z414F	GTGAACGTGCTGGCGCTGAACCT
Z414R	CGCTACCCGCCACTTATGCTCA
Z686R	TTGCCGCGAATTCGTGATAGTCC
Z687F	GTTACTTTGCGCATCGTTCACTCC
Z89F	CGCGTTGCTTTCGTACAGTTCCT

Z90R	GCAGCCAGAAGCGGAAGAAGACG
Z955F	ATGAGGTGATCGATGCCGTTTTTG
Z955R	CCTTCTCAAACCAGCAGCGTCGTG
Km1	CAGTCATAGCCGAATAGCCT
Km2	CGGTGCCCTGAATGAACTGC
Cm1	TTATACGCAAGGCGACAAGG
Cm2	GATCTTCCGTCACAGGTAGG
ZR-F	CTA**GTCTAGA**ATCCTGCTGCGGTGAGTAGT (XbaI)
ZR-R	CCC**AAGCTT**CGGCGTTTTATCACCAACTT (Hind III)
grrS-F	CTAG**TCTAGA**CGTTATTGTCTGGCGGGTCGTCAC (XbaI)
grrS-R	CCG**GAATTC**CGTTACAGGAAGCAGCGGAGAATG (EcoRI)
grrA-F	TTAAGCATCAGCGTGACCAG
grrA-R	GTATTCGCCTTTGGCATGTT
flhD-F	CCG**GAATTC**CAAGGCTGAAATTTCCCAAA (EcoRI)
flhD-R	CGC**GGATCC**AGAGCCAGAAGTCAGGCAGA (BamHI)
fliA-F	CCG**GAATTC**TAAAGCACGTCTGTGGCATC (EcoRI)
fliA-R	CGC**GGATCC**ACTCCTCCAGGGGAACATCT (BamHI)
amsG1	CGG**GGTACC**GTATCGCTTAAAGGGGAAACG(KpnI)
amsG2	CTA**GTCTAG**ACACCTGGAAAGCCATTAATCA(XbaI)

[a] Underlined nucleotides are restriction sites added and the restriction enzymes are indicated at the end of primers.

References

1. Yap MN, Yang C, Charkowski AO: **The response regulator HrpY of *Dickeya dadantii* 3937 regulates virulence genes not linked to the *hrp* cluster.** *Mol Plant-Microbe Interact* 2008, **21**: 304-314.
2. Kim D, Forst S: **Genomic analysis of the histidine kinase family in bacteria and archaea.** *Microbiology* 2001, **147**:1197-1212.
3. Galperin MY: **Structural classification of bacterial response regulators: Diversity of output domains and domain combinations.** *J Bacteriol* 2006, **188**:4169-4182.
