# Supplementary material for: Systems level analysis of two-component signal transduction systems in Erwinia amylovora: Role in virulence, regulation of amylovoran biosynthesis and swarming motility
Source: BMC Genomics. 2009 May 26;10:245. doi: 10.1186/1471-2164-10-245 (PMC2698875; doi:10.1186/1471-2164-10-245)
Supplement: Additional File 2 — Structural classification of histidine kinases, hypersensitive response of HrpXYS mutants in tobacco and schematic map of HrpXYS operon. The file contains two figures. Figure S1 shows structural classification of histidine kinases (HKs) based on phosphorylation sites (H-boxes); Figure S2 shows hypersensitive responses (HR) on tobacco for WT, ΔT3SS [47], hrpX, hrpY, hrpXY, and hrpXYS mutants (Figure S2A) and a schematic map of the HrpXYS operons (Figure S2B). [file 1471-2164-10-245-S2.pdf]

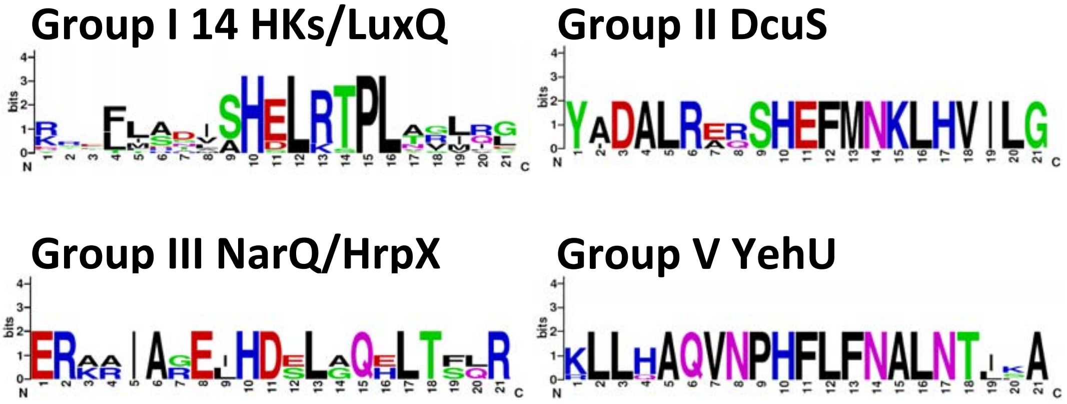

Figure S1. Structural classification of histidine kinases (HKs) based on phosphorylation sites (H-boxes). Each sequence logo is derived from alignment of proteins belonging to corresponding HK group and created by Weblogo (weblogo.berkeley.edu). The height of each residue is according to the level of conservation at that site so that degree of conservation is illustrated. Group I HKs contain an invariant Pro residue at position five downstream from an invariant His; Group II HKs possess a conserved Asn at position four downstream of His; Group III HKs have an ERxxxxxE motif; Group IV HKs consist of Pro residue proceeding the conserved His [Qian et al. *Mol Plant-Microbe Interact* 2008, **21**:151-161]. In Group I, 14 HKs are ArcB, BaeS, CpxA1, CpxA2, EnvZ, GrrS, KdpD, NtrC, PhoQ, PhoR, PmrB, RcsC, RstB and YfhK. For Group II and IV, DcuS (GenBank accession #BAE78127) and YehU (GenBank accession #AAA60489) of *Es. coli*, respectively, are included to generate the logo.

A

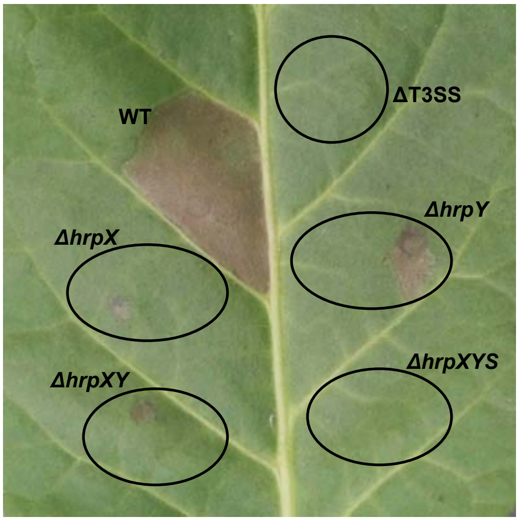

B

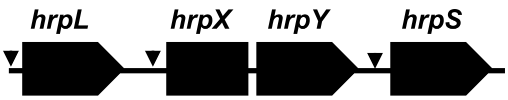

Figure S2. A. Hypersensitive response on tobacco for WT, ΔT3SS (T3SS island deletion mutant, deleted 33.5kb; as negative control), *hrpX*, *hrpY*, *hrpXY*, and *hrpXYS* mutants. Photos were taken at 24 hrs post inoculation. **B.** Schematic map of the *hrpLXYS* operons in *Erwinia amylovora*. Arrowheads indicate promoter. Draw not in scale.
